# An Iodine‐Vapor‐Induced Cyclization in a Crystalline Molecular Flask

**DOI:** 10.1002/anie.201601525

**Published:** 2016-04-06

**Authors:** Jane V. Knichal, Helena J. Shepherd, Chick C. Wilson, Paul R. Raithby, William J. Gee, Andrew D. Burrows

**Affiliations:** ^1^ Department of ChemistryUniversity of BathClaverton DownBathBA2 7AYUK; ^2^ School of Physical SciencesUniversity of KentCanterburyKentCT2 7NHUK

**Keywords:** cyclization, host–guest systems, iodine, porous networks, X-ray diffraction

## Abstract

A vapor‐induced cyclization has been observed in the host environment of a crystalline molecular flask (CMF), within which 1,8‐bis(2‐phenylethynyl)naphthalene (bpen), a diarenynyl system primed for cyclization, was exposed to iodine vapor to yield the corresponding indeno[2,1‐α]phenalene species. The cyclization process, unique in its vapor‐induced, solvent‐free nature, was followed spectroscopically, and found to occur concurrently with the displacement of lattice solvent for molecular iodine in CMF⋅0.75 bpen⋅2.25 CHCl_3_⋅H_2_O. The cyclization occurred under mild conditions and without the need to suspend the crystals in solvent. The ability of CMFs to host purely gas‐induced reactions is further highlighted by the subsequent sequential oxidation reaction of cyclized 7‐iodo‐12‐phenylindeno[2,1‐α]phenalene (ipp) with molecular oxygen derived from air, yielding 12‐hydroxy‐7‐iodo‐2‐phenylindeno[2,1‐α]phenalen‐1(12*H*)‐one (hipp).

The advent of crystalline molecular flasks^1^, ^2^ (CMFs) has provided a breakthrough in the crystallographic visualization of chemical processes and species.^3^, ^4^ This stems from the ability of the flexible coordination polymer in question, which is derived from 2,4,6‐tris(4‐pyridyl)‐1,3,5‐triazine (tpt) linkers and zinc halide nodes, to swell and contract upon solvent addition and loss,^5^ and undergo guest exchange in a single‐crystal‐to‐single‐crystal manner.^6^ Two strategies have been employed within CMF chemistry that employ either π–π interactions^7^ or complementary pore–guest sizes and shapes to localize guest molecules. The former strategy allows a modular approach to be employed by modifying the planar aromatic handle.^8^ This provides an anchor for postsynthetic modification (PSM) reactions that occur in the solid state and can be monitored crystallographically.^9^, ^10^, ^11^, ^12^, ^13^, ^14^, ^15^ In the absence of an aromatic handle, matching of the spatial dimension of the pore cavity is also a viable strategy to observe guests using crystallographic methods. This approach has been used to probe photoswitching,^16^ change conformational isomerization ratios,^17^, ^18^ observe unusual molecular interactions,^19^ and identify organic species,^20^, ^21^ with the notable advantage of requiring only nanogram to microgram quantities of the analyte.^3^

A range of chemical reactions have been observed within CMFs, including the conversion of amines into imines,^9^, ^10^ Huisgen cycloadditions,^11^ Diels–Alder reactions,^14^ and metal‐catalyzed methylation^12^ and bromination.^15^ All of these processes occurred upon immersing a CMF that contained a modified intercalated triphenylene moiety into solutions with appropriate chemical reactants. Only one example of a chemical reaction combining gas‐phase reagents and a CMF has been reported, and involved the conversion of a vinyl group into epoxidation/oxidation products with the aid of a radical initiator.^14^ This reaction, however, required loading of the CMF with 2,2′‐azobis(isobutyronitrile) and the use of forcing conditions, whereby an emulsion of crystals was heated at 80 °C for 24 hours in air. To the best of our knowledge, attempting chemical transformations in CMFs using gas‐phase reagents in the absence of solvents is unprecedented. Herein, we report an iodine‐vapor‐mediated cyclization of 1,8‐bis(2‐phenylethynyl)naphthalene (bpen) to 7‐iodo‐12‐phenylindeno[2,1‐α]phenalene (ipp; Scheme 1). This reaction makes use of a well‐known CMF with the composition [(ZnI_2_)_3_(tpt)_2_]⋅*x*(G), where G=guest.^9^, ^10^, ^11^, ^12^, ^13^, ^14^, ^15^ Cyclization of bpen to ipp in the presence of iodine vapor was found to occur quantitatively, under mild conditions, without complicated activation of the CMF, and in the absence of solvent.

**Scheme 1:**
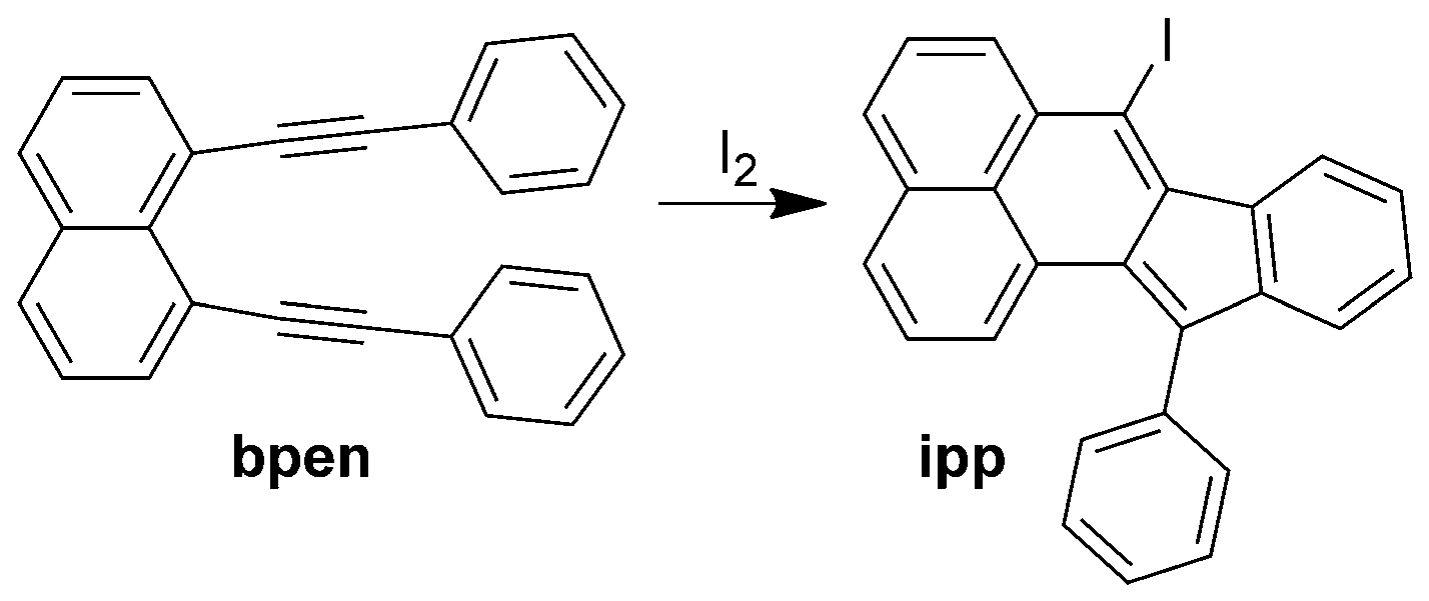
Cyclization of bpen to ipp mediated by molecular iodine.

Prior to loading bpen into the CMF, the solvated framework containing cyclohexane was first prepared by a solvent‐ exchange route from a precursor framework that had been synthesized from methanol and nitrobenzene, as reported by Fujita and co‐workers.^22^ Immersing the crystals loaded with cyclohexane into a concentrated solution of bpen in chloroform resulted in the crystals changing their color from colorless to yellow after two days. Analysis of the crystals by single‐crystal X‐ray crystallography determined a high bpen loading in the CMF, with elemental analysis revealing an occupancy of 0.75 and the remainder of the pores being occupied by chloroform (see below). The bpen guests could be observed in a crystallographic model along with two molecules of chloroform and a disordered molecule of water (Figure 1), giving an overall composition of [(ZnI_2_)_3_(tpt)_2_]⋅0.75 bpen⋅2.25 CHCl_3_⋅H_2_O (**1**). The chloroform hydrogen atom attached to C(65) is well aligned to participate in a weak C−H⋅⋅⋅π hydrogen bond to an alkyne group of bpen equidistant between C(56) and C(57), with an observed distance of approximately 3.45 Å. This interaction, coupled with a pair of π–π stacking interactions between the adjacent tpt ligand and the naphthalene ring (3.8 Å ring intercentroid distance) and alkyne group (3.8 Å ring–alkyne intercentroid distance) of bpen, serves to order the guests within the pores of **1**. The location of bpen guests within the CMF framework **1** is shown in Figure 2.

**Figure 1:**
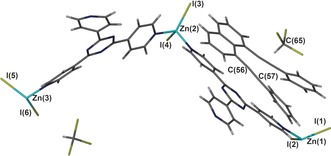
The asymmetric unit of **1** showing the CMF framework, which comprises three unique ZnI_2_ nodes and two tpt linkers, 0.75 equivalents of the bpen guest, and two molecules of chloroform. Disordered water has been removed for clarity.

**Figure 2:**
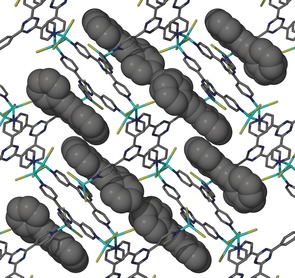
The location of the bpen guests (space‐filled molecules) within the CMF framework **1** (wire‐frame representation). Other guest solvents and hydrogen atoms have been omitted for clarity.

In the absence of bpen guests and solvent, the CMF possesses large 2D channels that propagate down the crystallographic *b* axis and the [101] direction.^5^ The channels are of a sufficient size to allow bpen to infuse into the crystals and contain protruding iodine atoms derived from the ZnI_2_ nodes, which provide a polarizing environment. These factors are expected to promote the facile adsorption of molecular iodine from the gas phase. To test this postulate, crystals of **1** were removed from their mother liquor, briefly dried in air, and placed in a small glass vial. This small vial was transferred into a larger vial containing solid iodine crystals and sealed, creating an atmosphere of iodine vapor in air that is capable of infusing into the crystals. Over several days the crystals changed color from yellow to black, coinciding with the uptake of molecular iodine. Single‐crystal X‐ray diffraction was performed on the black crystals after seven days, resulting in the identification of a structure containing molecular iodine and a region of highly disordered electron density, which was identified as the likely location of the guest cyclization product ipp: its presence was subsequently confirmed by NMR spectroscopy (see below). As expected, the structure of the host framework is comparable with that of **1**. It is reasonable to posit that the increase in disorder of the cyclized ipp guest in **2** relative to that of bpen in **1** may be due to the displacement of ordering solvent molecules by electronically diffuse molecular iodine. While the substantial disorder prevented the unambiguous identification of a single location for the ipp molecule, a structural model is presented in the Supporting Information in which the partially occupied, heavily restrained ipp molecule is included to illustrate its likely location within the pore of the ordered framework. The asymmetric unit, which includes the heavily restrained ipp molecule modelled within the region of highly disordered electron density, is shown in Figure 3. It must be stressed that the ipp molecule shown in Figure 3 serves only as a guide to the reader of its likely location, and does not constitute definitive crystallographic evidence. A model of the same structure excluding the ipp fragment is also presented for comparison (see the Supporting Information, Figure S3). In both structures of **2**, molecular iodine is included within the pores, again disordered over several identifiable and discrete positions. In each case, the total occupancy of I_2_ is between 1.75 and 2.1 molecules per [(ZnI_2_)_3_(tpt)_2_] unit, although the highly disordered nature of this material means that these values should be interpreted with caution. Although the overall quality of this structure was relatively low, further experiments were performed to unambiguously confirm the composition of **2**, which was determined to be [(ZnI_2_)_3_(tpt)_2_]⋅0.75 ipp⋅0.25 CHCl_3_⋅2 I_2_. This result was based on a combination of analytical techniques to determine the nature of the guest species (see below).

**Figure 3:**
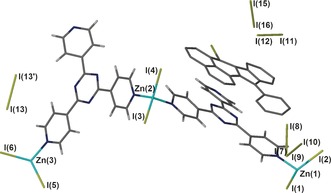
The asymmetric unit of **2** showing the likely location of the heavily restrained ipp guest molecule and disordered iodine molecules.

Single crystals of **2** were digested in [D_7_]DMF, and the resulting mixture was analyzed by ^1^H NMR spectroscopy (Figure S4). The spectrum contained no evidence of bpen; however, two new cyclized species were identified in a ratio of 1:1.6. The sample was subjected to column chromatography using hexane as the eluent to separate the two components. The major component eluted first and was determined to be the expected cyclization product ipp by ^1^H NMR spectroscopy (see below) as well as by X‐ray diffraction of the recrystallized product (Figure S3). The minor component was only isolable in trace amounts, but high‐resolution mass spectrometry (Figure S5) determined the unknown molecular ion to have a *m*/*z* ratio of 509.0005. This species was matched to an oxidized product of ipp (hipp; see Scheme 2) that is formed by (presumably subsequent) reaction with molecular oxygen ([*M*+Na]=509.0014). The conversion of ipp into hipp in the presence of molecular oxygen has been observed previously,^23^ albeit in solution and only after several weeks. This result demonstrates the ability of the CMF to host not only the iodine‐induced solvent‐free quantitative cyclization of bpen, but also consecutive gas‐mediated reactions in an unoptimized yield of more than 35 %, which greatly exceeds the trace amounts achieved in past reports.^23^

**Scheme 2:**
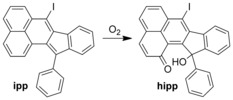
Oxidation of ipp to hipp mediated by molecular oxygen.

To better quantify the initial cyclization of bpen to ipp, the iodine infusion experiment was repeated in the inert argon atmosphere of a glove box. Single crystals held in this environment were analyzed by ^1^H NMR spectroscopy at intervals of 12 hours, 3 days, and 7 days (Figure 4). Digestion in [D_7_]DMF was undertaken in the glove box, and ^1^H NMR samples were protected from atmospheric O_2_ using Schlenk NMR tubes. Pure single‐crystalline ipp obtained after column chromatography was also dissolved in [D_7_]DMF in the absence of O_2_ and included as a reference. The results show that very little conversion (<5 %) had occurred after 12 hours despite the dramatic change in crystal color from yellow to black, suggesting that either iodine infuses into the crystal prior to mediating the cyclization, or that only the surface of the crystals has undergone reaction at this time. After three days, however, 85 % of the loaded bpen had cyclized to ipp, as determined by comparing the ratio of the residual *meta*‐phenyl resonances of bpen at 7.26 ppm to the new aromatic resonances of ipp at 7.05 ppm and 9.17 ppm. After seven days, bpen was no longer observable spectroscopically, and apart from residual tpt derived from digestion of the CMF and a signal at 8.39 ppm, which was assigned to chloroform, ipp was the dominant species observed. Interestingly, despite rigorous exclusion of oxygen from the point of iodine introduction, trace amounts of hipp were still observed in the spectra recorded after three and seven days. These findings can be attributed to the uptake of small amounts of molecular oxygen by **1** during the air‐drying step prior to transfer into the glove box. No evidence of hipp was observed after 12 hours, demonstrating the necessity of the iodine‐mediated cyclization step prior to hipp formation and supporting the conclusion that ipp is initially formed in the iodine‐gas‐mediation cyclization with subsequent transformation to hipp within the molecular flask in the presence of O_2_.

**Figure 4:**
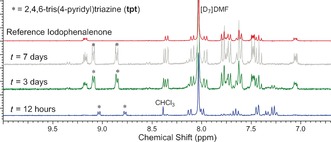
^1^H NMR spectra for CMF **1** digested in [D_7_]DMF. The degree of cyclization was evaluated at *t*=12 h, 3 days, and 7 days. The spectrum of 7‐iodo‐12‐phenylindeno[2,1‐α]phenalene (ipp) is included as a reference.

The cyclization of bpen to ipp requires a single iodine atom from molecular iodine and liberates a proton. The remaining iodide likely forms gaseous hydrogen iodide, which is liberated from the CMF under inert conditions. Under aerobic conditions, the hydrogen iodide may react further with molecular oxygen to give molecular iodine and water.

To better evaluate the degree of solvation within the CMFs **1** and **2**, thermogravimetric analysis was employed (Figure S6). Independent analysis of bpen prior to loading into the CMF showed that the guest is thermally stable up to 230 °C, after which time it loses approximately 50 % of its mass up to 400 °C. CMF **1** loses 8.1 % by mass in two steps up to a temperature of 200 °C, which is consistent with the crystallographic assignment of two chloroform molecules and one water molecule (predicted: 11.8 %). The iodine‐infused CMF **2** was found to have lost 19.9 % of its mass by 230 °C, which is consistent with the surmised presence of two equivalents of molecular iodine within the CMF (predicted: 20.0 %). The elemental analysis results for framework **1** were indicative of incomplete bpen loading, likely involving substitutional disorder of chloroform, with a ratio of 75 % bpen to 25 % CHCl_3_, providing matching C, H, and N percentages (Calcd [%]: C 32.79, H 1.92, N 7.95; found: C 32.76, H 1.68, N 7.95). This composition was used to fix the occupancy of the bpen molecule in **1** in the crystal structure, but it should be noted that it is possible to obtain variation between individual crystals with regard to guest loading, which is a known phenomenon in CMF systems.^22^ Elemental analysis performed on **2** was also consistent with a loading ratio of 0.75, with the remaining 0.25 assigned to residual chloroform based on ^1^H NMR spectroscopic and microanalytical evidence (Calcd [%]: C 27.22, H 1.45, N 6.83; found: C 27.01, H 1.29, N 7.34).

In conclusion, we have described gas‐mediated reactions that occur quantitatively within a CMF under mild conditions and without the need to activate the framework beyond the initial loading of reagents. Furthermore, the reaction occurred without any need to suspend the CMF in a solvent, simplifying methods for reaction monitoring. In the absence of air, bpen was converted into the cyclization product ipp by molecular iodine after seven days, as monitored by ^1^H NMR spectroscopy. Running the transformation in air resulted in a process of sequential gas‐mediated reactions, first iodine‐mediated conversion of bpen into ipp, followed by oxidation of ipp to hipp. The latter oxidation step proceeded with a conversion of more than 35 % after seven days without optimization, greatly improving on past solution‐based reports.^23^ To the best of our knowledge, this work represents the first report of sequential gas‐mediated reactions in a CMF system. These results expand the utility of CMFs from solution‐phase to gas‐phase reaction monitoring, and demonstrate their potential for undertaking sequential reactions in a “crystalline one‐pot flask”. This will be of great interest to the field of MOF‐based catalysis as well as for the development of gas‐sensing devices.

## Supporting information

As a service to our authors and readers, this journal provides supporting information supplied by the authors. Such materials are peer reviewed and may be re‐organized for online delivery, but are not copy‐edited or typeset. Technical support issues arising from supporting information (other than missing files) should be addressed to the authors.

Supplementary
