# Supplementary material for: An Iodine‐Vapor‐Induced Cyclization in a Crystalline Molecular Flask
Source: Angew Chem Int Ed Engl. 2016 Apr 6;55(20):5943–6. doi: 10.1002/anie.201601525 (PMC5071776; doi:10.1002/anie.201601525)
Supplement: Supplementary file 1 — Supplementary [file ANIE-55-5943-s001.pdf]

Angewandte Chemie
Eine Zeitschrift der Gesellschaft Deutscher Chemiker

Supporting Information

An Iodine-Vapor-Induced Cyclization in a Crystalline Molecular Flask

Jane V. Knichal, Helena J. Shepherd, Chick C. Wilson, Paul R. Raithby, William J. Gee, and Andrew D. Burrows**

anie_201601525_sm_miscellaneous_information.pdf

Table of Contents

1. Materials and Methods S2
2. Synthesis and Characterization S2
3. Crystallographic Modelling of **1**, **2** and ipp S3
4. Ratio of Cyclized products ipp and hipp in air S6
5. High-Resolution Mass Spectrum of hipp S7
6. Thermogravimetric Traces of bpen, **1** and **2** S8
7. References S9

1. Materials and Methods

Starting materials and solvents were purchased from commercial sources and used as received. The following literature reports were employed as part of this synthetic work: Synthesis of the bpen guest with matching to the reported characterisation;[S1] Synthesis and matching of characterisation for the MOF ligand tpt;[S2] Synthesis of the 'empty' MOF framework followed by loading of the pores with cyclohexane;[S3] and matching of characterisation of ipp and hipp with those present in literature reports.[S4] NMR spectra were recorded on a Bruker Advance 300 MHz Ultrashield NMR spectrometer. Infrared spectra were recorded on a PerkinElmer Spectrum 100 spectrometer equipped with an ATR sampling accessory. Abbreviations for IR bands are s = strong, m = medium, w = weak, br = broad. Elemental analyses (CHN) were performed on a CE-440-Elemental Analyzer (Exeter Analytical).

2. Synthesis and Characterization

*Synthesis of bpen loaded **1** ($[(ZnI_2)_3(tpt)_2]\cdot bpen\cdot 2CHCl_3\cdot H_2O$)*

Activated single crystals of the MOF framework loaded with cyclohexane were suspended in a concentrated solution of bpen dissolved in cyclohexane (0.5 mL) spiked with 1-2 drops of chloroform and subsequently sealed in an airtight vessel. After one week the formerly colourless crystals had adopted a yellow hue and a suitable single crystal was selected, suspended in Fomblin oil and mounted for crystallographic analysis. The bulk sample was briefly air dried before analysis or transference to the next reaction step. *Bulk characterisation*: FTIR $\bar{\upsilon}$ = 3051 (w), 2918 (m), 2849 (m), 1619 (w), 1568 (w), 1516 (s), 1487 (m), 1441 (m), 1422 (m), 1375 (s), 1345 (m), 1212 (m), 1059 (m), 1026 (m), 915 (w), 826 (m), 804 (s), 751 (s), 705 (m), 690 (m), 668 (m), 655 (s) cm^{-1}. $[(ZnI_2)_3(tpt)_2]\cdot 0.75bpen\cdot 2.25CHCl_3\cdot H_2O$ C_{calc}: 27.22, C_{found}: 27.01, H_{calc}: 1.45, H_{found}: 1.29, N_{calc}: 6.83, N_{found}: 7.34.

*Synthesis of iodine loaded **2** ($[[(ZnI_2)_3(tpt)_2]\cdot 0.75ipp\cdot 0.25CHCl_3)\cdot 2I_2$.)*

After brief air-drying, a bulk sample of bpen-loaded **1** was transferred into a glovebox and sealed within a vial containing a partitioned sample of iodine. After 7 days complete adsorption of iodine had occurred with concomitant cyclisation of bpen to ipp, yielding **2**. *Bulk characterisation*: FTIR $\bar{\upsilon}$ = 3051 (w), 2921 (m), 2851 (m), 1619 (w), 1573 (w), 1514 (s), 1439 (w), 1421 (m), 1371 (s), 1313 (m), 1233 (m), 1212 (m), 1157 (w), 1120 (w), 1057 (s), 1027 (s), 982 (w), 864 (w), 833 (w), 801 (s), 775 (s) 655 (s) cm^{-1}. $[(ZnI_2)_3(tpt)_2]\cdot 0.75ipp\cdot 0.25CHCl_3)\cdot 2I_2$ C_{calc}: 32.79, C_{found}: 32.76, H_{calc}: 1.92, H_{found}: 1.68, N_{calc}: 7.95, N_{found}: 8.47.

3. Crystallographic Modelling of 1, 2 and ipp

Single crystals of **1** and **2** were prepared as described above. In each case a suitable crystal was selected and mounted in oil on a SuperNova, EosS2 diffractometer; data were collected from a standard copper microfocus source. The crystal was kept at either 100(2) K or 150(2) K during data collection. Using Olex2,[S5] the structure was solved with the ShelXS[S6] structure solution program *via* Direct Methods and refined with the ShelXL[S7] refinement package using Least Squares minimisation.

Refinement details:

1

Some large residual electron density peaks are observed in the difference map of the final structure. They are all located close to the iodine atoms of the framework and are attributed to absorption effects caused by the copper X-rays used during the experiment. Cu radiation was deemed appropriate for data collection in an effort to resolve the light-atom guest located in the framework pore, which was of more interest to the manuscript than the framework itself.

In light of TGA and elemental analyses, the occupation of the bpen guest was fixed at 75%, and one water molecule was modelled as disordered over 3 positions.

One might expect the atomic displacement parameters to be significantly larger in a guest molecule than in the framework itself, reflecting the greater mobility of these fragments, and thus the usual procedure of fixing the ADPs and refining the occupancy directly was not considered reliable. However the ADPs of the guest bpen molecule were sensible when modelled with an occupancy of 75 %. Hence this value of 75 % obtained from XRD should be treated as a reasonable estimation rather than an absolute reflection of the loading percentage in that particular crystal.

2

The large pores contain a significant amount of disordered molecular iodine, ipp molecules and possibly solvent molecules. Some locations for partially occupied iodine molecules could be inferred from pairs of similarly sized electron density peaks separated by the expected I-I interatomic distance and were modelled with equal ADPs of both atoms and the occupancy was allowed to refine freely. A large single peak that did not appear to be attributable to molecular iodine was ascribed to one possible location of the Iodine atom on the ipp molecule. However, no discrete carbon positions that can be unambiguously attributed to the ipp were found in the difference map. Consequently the rest of the atoms in that molecule were placed at calculated positions and refined with partial occupancy and heavily restrained and constrained. The aim of providing this model is merely to demonstrate one possible location of the ipp product molecule and show that it fits within the available pore space. This model is referred to as 2-ipp in the following crystallographic data tables.

For comparison we also present a model of the same data excluding the ipp molecule in a separate cif (denoted 2-no_ipp). Neither model is truly satisfactory in terms of locating the ipp guest molecule

in this porous material. It reflects a situation in which the molecule is not ordered, a common feature of porous, guest-containing MOFs.

In both cases large residual electron density peaks are observed in the region of the pore, and still cannot be unambiguously assigned to specific molecules. In light of the interest in the present publication being the reactions occurring within the pores, use of the SQUEEZE routine (or similar solvent masking techniques) was not deemed useful. However, the NMR, TGA, elemental analysis and XRD of the recrystallized product prove unambiguously that the bpen molecule is converted into ipp within the framework pore.

ipp

A new polymorph of ipp was isolated during the course of this work (Figure S1). A previous polymorph of this material is known and has been characterized elsewhere.[S8]

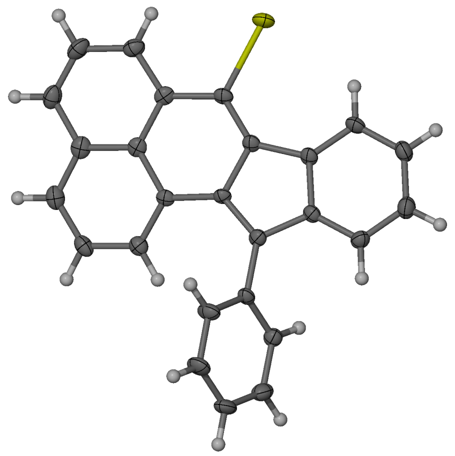

Figure S1. Molecular structure of ipp. Atom legend: dark grey = carbon, light grey = hydrogen, yellow = iodine). Ellipsoids are shown with 50% probability.

Table S1 Crystal data and structure refinement for **1**, **2** and ipp.

Identification code	1	2-ipp	2-no_ipp	ipp
Empirical formula	$C_{57.5}H_{38}Cl_6I_6N_{12}OZn_3$	$C_{39.85}H_{26}I_{8.33}N_{12}Zn_3$	$C_{36}H_{26}I_{7.7}N_{12}Zn_3$	$C_{26}H_{15}I$
Formula weight	2083.21	1925.85	1799.93	454.28
Temperature/K	100(2)	150.0(1)	150.0(1)	150.0(1)
Crystal system	monoclinic	monoclinic	monoclinic	monoclinic
Space group	*C*2/*c*	*C*2/*c*	*C*2/*c*	$P\,2_1/c$
a/Å	33.6889(3)	34.5922(11)	34.5922(11)	11.84806(16)
b/Å	14.64640(11)	14.8141(5)	14.8141(5)	18.3672(2)
c/Å	31.9521(3)	31.4266(13)	31.4266(13)	8.34925(12)
β/°	99.1492(9)	100.790(3)	100.790(3)	98.0542(13)
Volume/$Å^3$	15565.3(2)	15819.9(10)	15819.9(10)	1799.01(4)
Z	8	8	8	4
$\rho_{calc}g/cm^3$	1.778	1.617	1.511	1.677
μ/mm^{-1}	22.002	26.803	24.859	14.018
F(000)	7880.0	7044.0	6593.0	896.0
Crystal size/mm^3	0.1717 × 0.1101 × 0.0732	0.373 × 0.2048 × 0.1624	0.373 × 0.2048 × 0.1624	0.4 x 0.3 x 0.1
Radiation	CuKα (λ = 1.54184)	CuKα (λ = 1.54184)	CuKα (λ = 1.54184)	CuKα (λ = 1.54184)
2Θ range for data collection/°	8.378 to 144.53	10.642 to 132.07	9.822 to 132.07	4.4722 to 71.9559
Index ranges	−41 ≤ h ≤ 41<br>−17 ≤ k ≤ 18<br>−39 ≤ l ≤ 39	−39 ≤ h ≤ 40<br>−10 ≤ k ≤ 17<br>−22 ≤ l ≤ 36	−39 ≤ h ≤ 40<br>−10 ≤ k ≤ 17<br>−22 ≤ l ≤ 36	−14 ≤ h ≤14<br>−22 ≤ k ≤17<br>−10 ≤ l ≤10
Reflections collected	139686	25254	25261	10446
Independent reflections	15233<br>[R_{int} = 0.0498<br>R_{sigma} = 0.0238]	13382<br>[R_{int} = 0.0506<br>R_{sigma} = 0.0547]	13386<br>[R_{int} = 0.0506<br>R_{sigma} = 0.0547]	3533<br>[R_{int} = 0.0318<br>R_{sigma} = 0.0459]
Data/restraints/parameters	15233/206/809	13382/72/631	13386/3/565	3533 / 0 / 244
Goodness-of-fit on F^2	1.022	1.597	1.778	1.025
Final R indexes [I>=2σ (I)]	R_1 = 0.0586<br>wR_2 = 0.1531	R_1 = 0.1326<br>wR_2 = 0.3651	R_1 = 0.1521<br>wR_2 = 0.4048	R_1 = 0.0304<br>wR_2 = 0.0790
Final R indexes [all data]	R_1 = 0.0644<br>wR_2 = 0.1580	R_1 = 0.1488<br>wR_2 = 0.3913	R_1 = 0.1687<br>wR_2 = 0.4302	R_1 = 0.0319<br>wR_2 = 0.0809
Largest diff. peak/hole / e $Å^{-3}$	2.85/−2.60	5.02/−2.69	8.91/−2.83	0.635/−0.963

CCDC 1450578-1450581 contains the supplementary crystallographic data for this structure. These data can be obtained free of charge from The Cambridge Crystallographic Data Centre via www.ccdc.cam.ak.uk/data_request/cif.

4. ^{1}H-NMR Determination of the Ratio of Cyclized Products under air

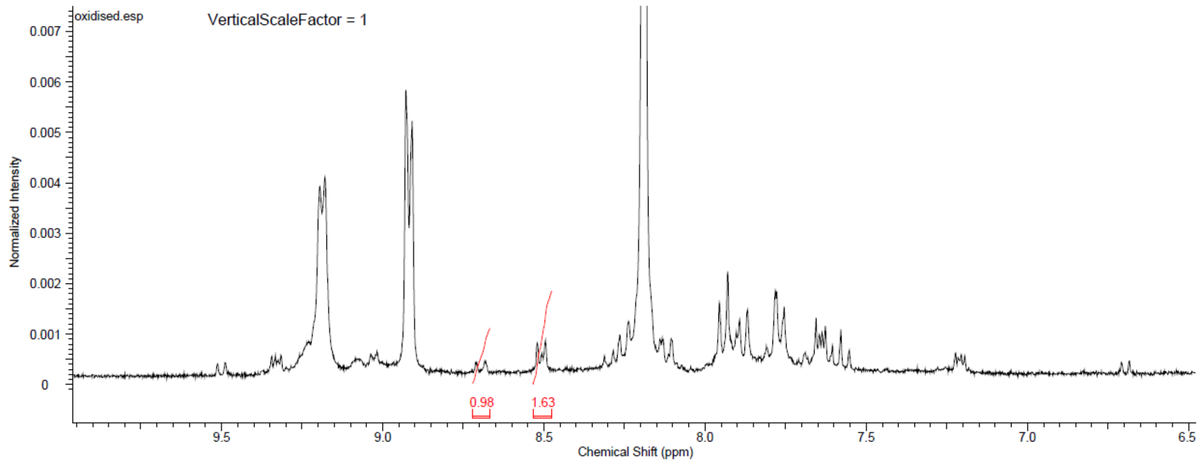

The ratio of ipp to hipp was made after a solid sample of crystalline **1** was sealed in an atmosphere or air with a partitioned sample of crystalline iodine. After seven days the crystals were digested in DMF-d_7 and the resonances of two non-overlapped aromatic protons (δ = 8.51 (ipp) and 8.70 (hipp) ppm) and compared. Prominent signals at *ca* 8.2, 8.9 and 9.2 correspond to DMF-d_7 and the aromatic signals from tpt, respectively.

5. High-Resolution Mass Spectrum of hipp

Confirmation of Expected Formula

Sample-ID	ra_wg_MOFF2	Submitter	Will Gee
Analysis Name	ra_wg_MOFF2_344866_40_01_49390.d	Supervisor	Paul Raithby
Method used	Confirm Formula Positive 50to1500 loop inj.m	Acquisition Date	09/09/2015 13:38:00
Ionisation Mode	positive electrospray (ESI)		

+MS, 1.0-1.3min #(47-60), -Spectral Bkgrnd

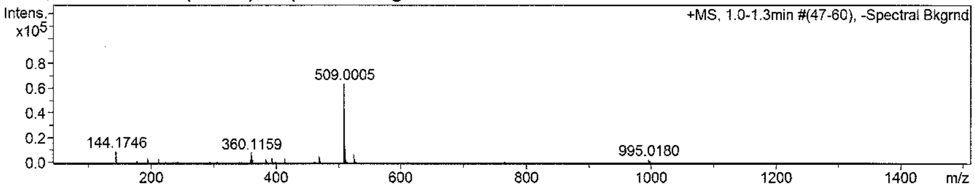

#	m/z	I	I %	Area	S/N
1	144.1746	9418	14.9	210	5091.6
2	195.1856	4072	6.4	172	1082.3
3	212.1194	3434	5.4	145	747.0
4	360.1159	7926	12.5	606	530.3
5	393.2994	4212	6.7	344	237.0
6	413.2757	3955	6.3	151	207.5
7	469.0104	5013	7.9	496	221.4
8	509.0005	63272	100.0	6041	3060.9
9	510.0058	17350	27.4	1789	843.8
10	524.9957	6844	10.8	801	361.7

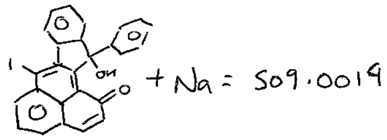

Generate Molecular Formula Parameters

Charge	Tolerance	SearchRadius	H/C Ratio min.	H/C Ratio max.	Electron Conf.	Nitrogen Rule	sigma limit
positive	10 ppm	0.05 m/z	0	3	both	true	0.05

Expected Formula C26 H15 I O2 **Adduct(s):** H, Na

#	meas. m/z	theo. m/z	Err[ppm]	Sigma	Formula
1	509.0005	509.001442	0.80	0.0054	C 26 H 15 I 1 Na 1 O 2

Note: Sigma fits < 0.05 indicates high probability of correct MF, and mass accuracy of 5ppm or better is generally acceptable for publication

Department of Chemistry Mass Spectrometry Service printed: 09/09/2015 13:40:38 Page 1 of 1

6. Thermogravimetric Traces of bpen, 1 and 2

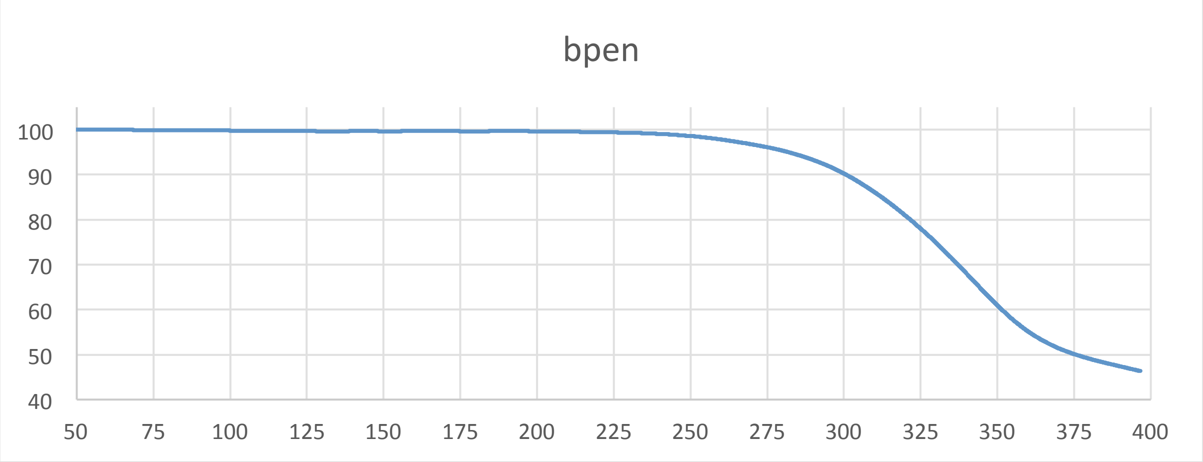

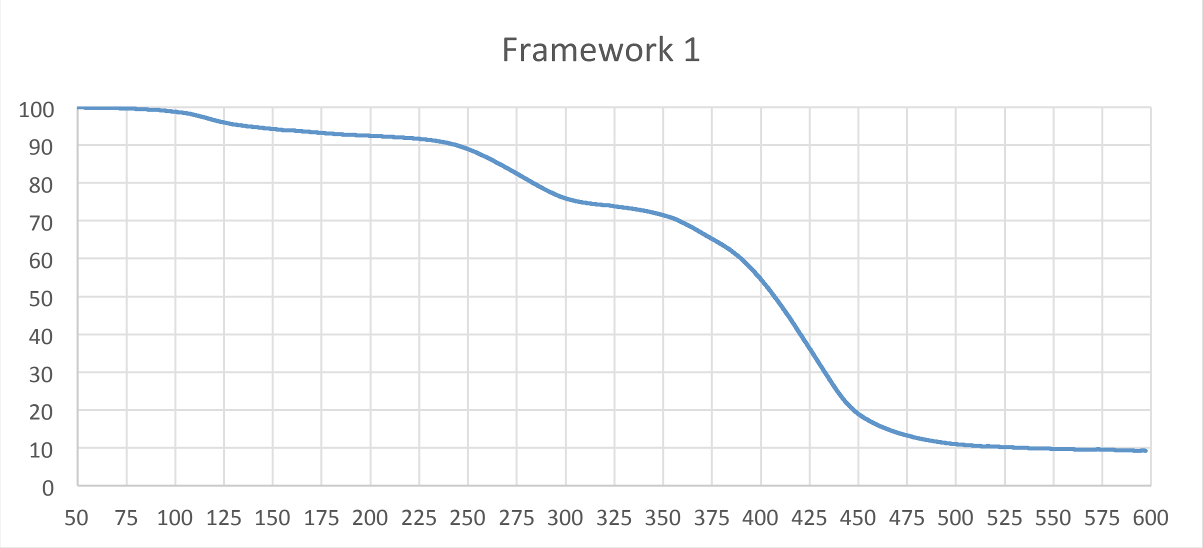

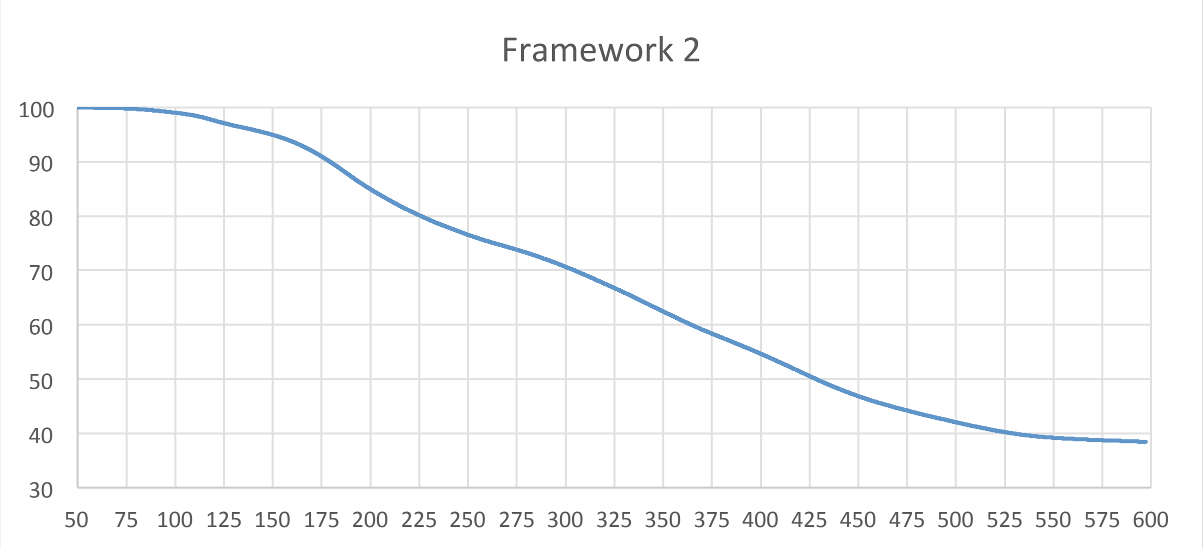

7. References

S1: Wu, Y.-T.; Hayama, T.; Baldridge, K. K.; Linden, A.; Siegel, J. S. *J. Am. Chem. Soc.* **2006**, *128*, 6870.

S2: Herrera, A.; Martínez-Alvarez, R.; Ramiro, P.; Chioua, M.; Chioua, R. *Synthesis*, **2004**, *4*, 503.

S3: Inokuma, Y.; Yoshioka, S.; Ariyoshi, J.; Arai, T.; Fujita, M. *Nat. Protoc.* **2014**, *9*, 246.

S4: Huang, X.; Zeng, L.; Zeng, Z.; Wu, J. *Chem. Eur. J.* **2011**, *17*, 14907.

S5: Dolomanov, O. V.; Bourhis, L. J.; Gildea, R. J.; Howard, J. A. K.; Puschmann, H. *J. Appl. Cryst.* **2009**, *42*, 339.

S6: Sheldrick, G.M. *Acta Cryst.* **2008**, *A64*, 112.

S7: Sheldrick, G.M. *Acta Cryst.* **2015**, *C71*, 3.

S8: X. Huang, L. Zeng, Z. Zeng, J. Wu, *Chem. Eur. J.* **2011**, *17*, 14907.
